# Supplementary material for: Molecular Relay Stations in Membrane Nanotubes: IRSp53 Involved in Actin-Based Force Generation
Source: Int J Mol Sci. 2023 Aug 23;24(17):13112. doi: 10.3390/ijms241713112 (PMC10487789; doi:10.3390/ijms241713112)
Supplement: Supplementary file 1 [file ijms-24-13112-s001.zip › ijms-2516089-supplementary.pdf]

Supplementary Material

Molecular relay stations in membrane nanotubes: IRSp53 involved in actin-based force generation

Tamás Madarász [1], Brigitta Brunner [2], Henriett Halász [1], Elek Telek [1], János Matkó [3], Miklós Nyitrai [1] and Edina Szabó-Meleg [1,*]

[1] Department of Biophysics, Medical School, University of Pécs, H-7624 Pécs, Hungary

[2] Institute of Biology, Faculty of Sciences, University of Pécs, H-7624 Pécs, Hungary

[3] Department of Immunology, Faculty of Science, Eötvös Loránd University, H-1117 Budapest, Hungary

* Correspondence: edina.meleg@aok.pte.hu

Keywords: actin; fluorescence microscopy; IRSp53; membrane nanotube; protein−protein interactions

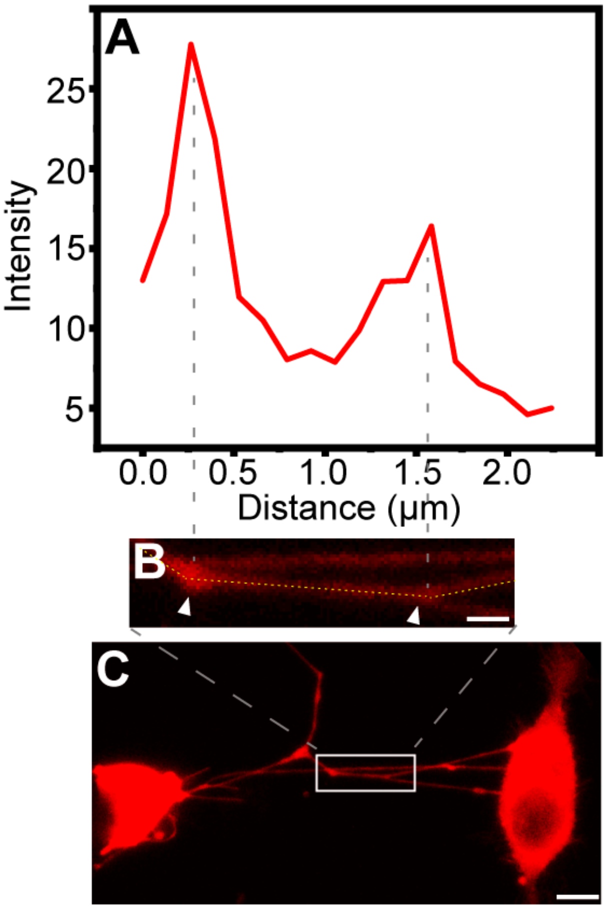

Supplementary Figure S1. Intensity profile along a branched NT showing an increase in the grey scale value at the position of IRSp53 accumulations. (**A**) Line scan analysis showing the intensity values in the function of the distance. (**B,C**) The analysis was performed along the NT (marked with a yellow dotted line), where analysed IRSp53 accumulation puncta are symbolized with white arrowheads. The area enclosed by the white frame was magnified. Scale bars: 3 μm (**B**) and 10 μm (**C**), respectively.

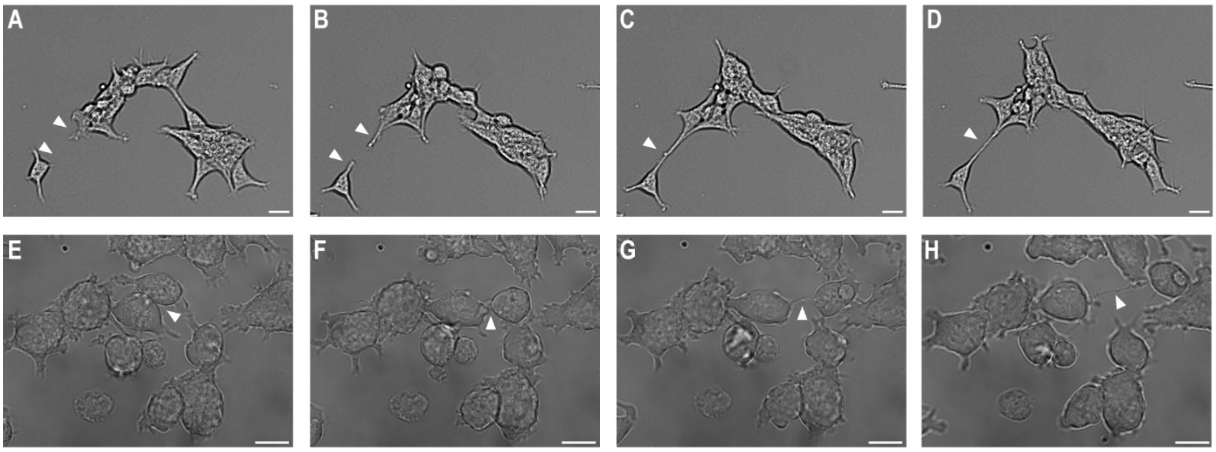

Supplementary Figure S2. Typical mechanisms of NT formation (making contact and keeping contact). (**A–D**) Kidney cells. (**E–H**) B cells. Arrowheads show the processes of NT initialisation. Scale bars: 10 μm.

	Parameters	COS-7		A20	
		I-BAR	IRSp53	I-BAR	IRSp53
Filopodia	Frequency of filopodia	increase	increase	increase	increase
	Length of filopodia	increase	increase	increase	increase
NTs	Frequency of NTs	increase	increase	no effect	increase
	Length of NTs	no effect	increase	no effect	increase
	Thickness of NTs	decrease	decrease	decrease	no effect
	Frequency of NT branching	increase	increase	increase	increase
	Length of NT branching	increase	increase	not reported	not reported

Supplementary Table S1. The summary of the effect of the overexpression of I-BAR and IRSp53 on the NTs and filopodia of the examined cell types. The overexpression of the I-BAR domain and the full-length IRSp53 protein caused different effects on the cell projections of COS-7 and A20 cells.

Supplementary Video S1. Vesicular transport in an NT of mCherry-IRSp53 transfected COS-7 cells. IRSp53: red, vesicles: light grey.

Supplementary Video S2. Vesicular transport in an NT of mCherry-IRSp53 transfected A20 cells. IRSp53: red, vesicles: light grey.

Supplementary Video S3. Optical slicing reveals that filopodia and NTs are located in different focal planes. XZ reconstruction of mCherry-IRSp53 transfected COS-7 cells. (CLSM, 63×). Filopodia are attached to the surface of the culture dish, and NT is stretched between two cells.

Supplementary Video S4. Time-lapse recording shows that NT is freely floating in the culture medium. Filopodia are attached to the substrate, only their tips are allowed to move freely.

Supplementary Video S5. Latrunculin A treatment of COS-7 cells induced the occurrence of retraction fibres. The cell bodies are round. Only retraction fibres attach to the bottom of the culture dish, and NTs that connect distant cells are floating in the media.
